# People, posts, and platforms: reducing the spread of online toxicity by contextualizing content and setting norms

**DOI:** 10.1007/s44204-022-00042-2

**Published:** 2022-08-19

**Authors:** Isaac Record, Boaz Miller

**Affiliations:** 1 Lyman Briggs College, Michigan State University, East Lansing, MI USA; 2 Department of Management Information Systems, Zefat Academic College, 11 Jerusalem St, 684703 Safed, Israel

**Keywords:** Social epistemology, Testimony, New media, Sharing, Norm of assertion, Technological possibility

## Abstract

We present a novel model of individual people, online posts, and media platforms to explain the online spread of epistemically toxic content such as fake news and suggest possible responses. We argue that a combination of technical features, such as the algorithmically curated feed structure, and social features, such as the absence of stable social-epistemic norms of posting and sharing in social media, is largely responsible for the unchecked spread of epistemically toxic content online. Sharing constitutes a distinctive communicative act, governed by a dedicated norm and motivated to a large extent by social identity maintenance. But confusion about this norm and its lack of inherent epistemic checks lead readers to misunderstand posts, attribute excess or insufficient credibility to posts, and allow posters to evade epistemic accountability—all contributing to the spread of epistemically toxic content online. This spread can be effectively addressed if (1) people and platforms add significantly more context to shared posts and (2) platforms nudge people to develop and follow recognized epistemic norms of posting and sharing.

The unchecked spread of epistemically toxic content online has seen a surge of handwringing attention from journalists and scholars (including philosophers) in the last decade, especially since the 2016 US presidential election. Often discussed under the banners of “misinformation” or “fake news,”^1^ we adopt the general term “epistemically toxic content” to highlight the purported problematic feature of such content: it damages our epistemic environment.^2^ This damage has been supposed to be at the root of crises for democracy, news media, culture, and so on. However, as we will argue, neither the prominence nor the worst effects of epistemically toxic content are inevitable: they are consequences of the specific social and technical systems currently in operation. We present a novel three-part model of individual people, online posts, and media platforms. We argue that online sharing constitutes a distinctive communicative act, governed by a dedicated non-epistemic norm. We identify several causes for the lack of a stable norm of sharing, most prominently online context collapse. We argue that an unstable and unclear norm of sharing, which lacks inherent epistemic checks, contributes to the spread of epistemically toxic content. It also leads readers to misunderstand posts or attribute excess or insufficient credibility to posts, and it allows posters to evade epistemic accountability for harmful posts.

We argue that the spread of epistemically toxic content online can be effectively addressed if (1) people and platforms significantly add more contextual information to shared posts and (2) platforms nudge people to develop and adhere to recognized epistemic norms for posting.

## People, posts, and platforms

This section proposes a threefold model of the media environment. First, the media environment consists of *people*, who create, interpret, and interact with *posts* using operations the *platform* affords and a corresponding conceptual idea of how and why to use the platform. Second, it consists of *posts*, the atomic units of content, which may be self-contained or refer to other content. *People* create and interpret posts according to their motivations and their beliefs about the media, and decide which *platform*-provisioned operation(s) to perform. Third, it consists of *platforms*, which supply *people* with content in the form of a set of *posts*, and afford people operations to perform on those posts, such as reporting, “reactions” like “likes” or smiley faces, reposting, replying, making a new post, or simply scrolling.

We understand the connection between people, posts, and platform in terms of “technological possibility” (Record, 2013; Miller and Record, 2013; Record and Miller, 2018) or “affordance” (Davis, 2020; Kiran and Verbeek 2010), which depends on access to both the conceptual and material means to carry out a course of action, and its cousin, practicability. Where technological possibility sets a theoretical limit for what can be accomplished given access to technology, in practice, our range of activities is narrowed to those we consider practicable given our goals. New technology makes previously impracticable actions practicable. Such enlivened possibilities effectively change existing standards of moral and epistemic responsibility since our normative expectations for responsible conduct are tied to what a subject can do *in practice* to bring about desirable results or prevent undesirable ones (Record, 2013; Miller and Record, 2013).

Social media platforms provide users with a limited set of constantly changing operations to create, read, and engage with posts. Six material properties of posts are especially relevant for understanding people’s engagement: modularity, algorithmic surfacing, adjacency, archivability, modifiability, and accessibility.^3^ The first four properties help determine what platform users see when they log on. Many platforms divide content into modules called posts. Modularity contributes to the problem of context collapse discussed in Sect. 3. The largest platforms surface posts algorithmically. The detailed operation of these algorithms is secret (cf. Miller and Record, 2013), but it appears that in pursuit of “engagement” or attention, they amplify some content to the point it becomes epistemically toxic.^4^ The algorithm’s fixation on engagement leads to unintended consequences for other dimensions of the media landscape, such as truth. While platforms allow threading or connecting related content, each thread is placed adjacent to unrelated partners. Where many traditional media publications distinguish different kinds of content, separating commentary from news, for example, social media treats all content impartially, algorithmically flattening them to one preeminent dimension: engagement scores.^5^ Posts are also archivable, meaning they can be resurfaced and shown out of time.

The other two properties of social media platforms help shape what people can do on the platform. Posts are accessible and modifiable: nearly anyone can participate in light or heavy ways. In addition to reading and creating posts, people can report a post, apply a “reaction” like “like” or a smiley face, repost, or reply. Modifiability is a key feature of Web 2.0, which allows participatory culture to scale. Participatory culture assumes that the audience has a great deal of autonomy to act within the media space, unlike some traditional media forms, where the audience has been assumed to be passive.^6^ Some of these ways of engaging with content are extremely lightweight as compared to the relatively heavyweight correctives some critics suggest, which makes an important difference in which activities are practicable, as we will see in Sect. 5.

## Media ideologies

In addition to the material means of peoples’ interactions with social media platforms, conceptual means of engaging also shape which activities are more or less practicable for a particular person. Conceptual means center on what Ilana Gershon (2010) calls a “media ideology”: a person’s system of beliefs about how a medium communicates and structures communication. People conceive of Twitter, text messages, voice calls, and video chats differently partly for the different actions they afford. But media ideology is not just about material differences. People use video conferencing differently with friends than colleagues, or may use personal and work email accounts differently. People use identical technologies differently in different contexts because they have different media ideologies about their use within those contexts.

Gershon identifies several dimensions on which people evaluate media, including *formality*—for example, young people think of email as *formal*, while older people think of email as *informal*—and *similarity* to other media or face-to-face conversation. Media ideology helps us understand the difference between asking someone for coffee on Facebook (a request for a date) versus LinkedIn (a request for an informational interview). Media ideologies must be at least partially shared for communication to succeed. Media ideologies can also be highly personal:

*The Break*: Halle and Doug have been dating for a few weeks. They have been texting jokes to each other. Suddenly Doug texts Halle a serious breaking-up message. Halle does not interpret Doug’s message correctly, because it violates their shared media ideology about texting and contradicts her second-order information (Gershon 2010, 20–21).

Gershon writes that there is nothing materially inherent in a medium to make a certain media ideology about it more correct (2010, 21). Gershon is right to credit people with autonomy in meaning-making. Twitter users created hashtags as an informal but searchable tag for their posts (using an octothorpe, “#,” to signify the tag) before the platform adopted them into the official feature set. Twitter and SMS users escape the character limit by threading multiple posts together. It is said, disparagingly, “you just can’t be formal in 140 characters.” This is not true: telegrams and certain newsprint stories, such as death notices, have a long tradition of formality and brevity. Nevertheless, current shared media ideologies may well preclude formality in brief media.

That said, unlike Gershon, we think that a medium’s technological features such as message length do *restrict* possible media ideologies. For example, when the only possible reaction to a Facebook post was like, users felt unease “liking” a sad post, such as a death announcement. The introduction of the “sad” reaction solved this problem and settled as the appropriate reaction to a mourning post. We stress this because our solution to the spread of epistemically toxic content makes use of platforms’ ability to restrict users’ possible media ideologies.

Through qualitative research, Gershon has found that new-media ideologies significantly differ between people and are not shared among people of the same class, generation, race, or other demographic groupings. One reason for this variability is that new media are constantly changing feature sets, not allowing media ideologies to stabilize. Furthermore, a change to the conceptual idea about a medium or its material properties can disrupt the equilibrium, triggering a new round of conceptual and material adjustments.^7^ Importantly, people are unaware that other people do not share their media ideologies.^8^

## Context collapse

In the previous sections, we characterized the interrelations between people, platforms, and posts using the concepts of technological possibility and media ideologies. We argued that technological possibilities partly shape practicable actions and the normative standards associated with them, as well as users’ media ideologies. One such novel possibility afforded by social media is posting to wide, multiple audiences, including strangers. Social media also allows members of different audiences to mutually interact in the same post. Media technology currently does not allow people to effectively distinguish or separate between different audiences or identify which person belongs to which crowd. In this section, we discuss these novel possibilities and in the next section their implications to the spread of epistemically toxic content.

Alice Marwick and danah boyd (2011) introduce the term “context collapse” to describe the collapse of multiple conversation contexts into one context on social media:

We present ourselves differently based on who we are talking to and where the conversation takes place – social contexts like a job interview, trivia night at a bar, or dinner with a partner differ in their norms and expectations. The same goes for socializing online. Participants have a sense of audience in every mediated conversation, whether on instant messenger or through blog comments. This audience is often imagined and constructed by an individual in order to present themselves appropriately, based on technological affordances and immediate social context (2011, 114–115).

On Facebook and Twitter, a single post has multiple audiences. Some of its readers may know the poster personally outside the platforms, some may know her only from the platform, and some may not know her at all. Audience members may come from different geographical locations, speak different languages, and belong to social circles and clusters (work, family, neighborhood) that do not mix offline.

To correctly understand a post, its audience must fill in information gaps. What is this post about? Is it serious or a joke? Is the poster’s stance toward the post positive, negative, or neutral? To answer such questions, audience members draw on their own knowledge of the poster and the post’s subject matter, their background assumptions, and experience. Context collapse means that audiences with different media ideologies, background knowledge, assumptions, and expectations find themselves in the same media context, reading the same post and mutually interacting.

When context collapses, a post may become scandalous. The people who posted it or appear in it lose control of its interpretation and spread. Ordinary people, who have allegedly violated a social norm, may become the target of disproportional shaming.

*In Joke*: In 2013, a British PR woman, who had about 170 followers, tweeted “Going to Africa, Hope I don’t get AIDS. Just kidding, I’m white!” just before she boarded a flight from London to Cape Town. She intended this tweet as a private joke among her friends about her white privilege. She counted on the followers not to interpret the joke as racist. But by the time she landed, she had become the target of mass shaming, to the extent people were stalking her in the airport. Her tweet became viral. It was shared by millions, who denounced her as racist. She was fired, and could not find another job, because her tweet continued to appear when people Googled her name (Ronson 2015, 63–74). This is just one example of many with a similar pattern.^9^

*In Joke* illustrates how context collapse may cause a joke to go horribly wrong when it escapes its intended context. Context collapse may also work the other way by allowing posters to evade epistemic and moral responsibility for their posts, by pretending that their post was a misunderstood joke:

*Waldorf Astoria*: In August 2020, Shimon Riklin, an Israeli right-wing journalist, tweeted that he was denied access to the Waldorf Astoria Jerusalem Hotel because he was not part of the protests against PM Netanyahu in Jerusalem at that time. His tweet was widely shared. Several hours later, the hotel spokesman tweeted that the hotel CCTV footage revealed that Riklin had not tried to enter the hotel. In response, Riklin tweeted that his original tweet was a “joke,” and accused the hotel management of lacking a sense of humor. Riklin later deleted his original tweet.^10^

Requiring posters to completely specify their meaning in total detail is not practical, because much of what we communicate is not in what we say. According to Paul Grice (1989), many of the things we learn from what other people say is not explicitly said by them. Rather, we legitimately infer things (“conversational implicatures”) according to conventional maxims. Consider the following exchange:

*Pit Stop*:

Passenger: “I’m hungry.”

Driver: “There’s a gas station two kilometers ahead.”

The passenger would be warranted inferring that they will stop at that gas station, where food will be available. Spelling this out in fine detail would feel pedantic, even insulting, to the passenger.

In different contexts, people need to be more or less certain of the information they share, more or less detailed, hew more or less to the agreed upon topic, etc. When context collapses, the clues for assessing implicatures are gone, raising the likelihood of mistakes. This collapse of known implicatures occurs online in part because of the structuring of content into atomic posts that are placed adjacent to one another in a feed, in part because of the behaviors of posters online, and in part because the content of posts themselves rarely conveys the details of its originating context.

## The norm of sharing

After having introduced the theoretical framework, in this section, we discuss toxic content spread through posting and sharing, which we address as norm-governed actions.

Many actions are norm-guided. Dress norms dictate what people wear to an academic conference, a business meeting, or the beach. Norms determine the order in which customers are served at the supermarket checkout or board a bus. Conversational norms determine the appropriate tone of voice and choice of words in different circumstances. Norms vary between periods, cultures, places, groups, and even households. Norms can be violated. One may violate a norm intentionally, to attract attention, to protest against it, or just because one cannot be bothered to follow it, or unintentionally, not knowing that it prevails.

What kind of norm governs sharing? A plausible starting point is to look to ordinary conversational norms. For example, we acquire many beliefs from others’ say-so. Assertion, the speech act of telling, informing, or offering testimony,^11^ is guided by an *epistemic* norm, namely, a norm that pertains to the truth, validity, or certainty of the assertion. This norm is evident when we expect people to tell the truth, or qualify their claims when they are unsure. Non-epistemic norms, such as politeness, also govern assertion.

According to Timothy Williamson (2000, Ch. 11), knowledge is the norm of assertion: one should assert only what she knows. Williamson’s argument is straightforward: knowledge is the norm of assertion because assertion is the speech act whose function is to transmit knowledge. This is why an assertion is challenged by “how do you know?.”

But there are problems with Williamson’s hypothesis. Sometimes people legitimately assert although they do not know, apparently without violating the norm. Such assertions may be demanded or encouraged by a norm. The literature is now filled with cases in which people seem to legitimately assert claims that fall short of knowledge, and with alternative suggested formulations of the norm of assertion (Benton, 2020). The norm of assertion has also been studied empirically, and the results seem inconclusive.^12^

A possible explanation of the plurality of such cases is that the norm of assertion is contextual. Making this case, Goldberg (2015. Ch. 9) notes that philosophers normally assert things they do not know, do not take themselves to know, and are not taken by their audience to know. So it is in politics and religion, and as Dang and Bright (2021) argue, in science.

In this paper, we endorse the view that the norm of assertion is *contextual*, or at least, if there is a general norm of assertion, it is overridable in specific contexts. Taking a cue from Goldberg (2015, Ch. 10), we roughly characterize a contextual norm of assertion as follows:

*Contextual-norm-of-assertion:* assert that p only if p satisfies your audience’s information needs in terms of quality.

If the norm of assertion is contextual, context collapse and media ideologies explain its instability. Context collapse means there is no single audience with a distinctive set of information needs. Epistemic expectations may vary significantly among readers interpreting the same assertion made in the same post. When users’ perception about the correct norms that operate in a context are affected by the medium in which the assertion (or other communicative act) is made, then the norm of assertion, or beliefs about it, are part of users’ media ideologies.

The following example illustrates how the absence of stable epistemic norms allows users to evade responsibility for their posts:

*Election photo*: On September 19, 2019, Israeli election day, Yair Netanyahu, son of PM Benjamin Netanyahu, retweeted a post that urged Netanyahu supporters to vote, claiming that voting rates in the Arab sector were unusually high (Israeli-Arab voters typically oppose PM Netanyahu). The post was accompanied with a photo of Arab-looking people standing in line for ballots. It turned out, however, that the photo was taken in Turkey. Because the photo is not related to the claim, some may regard this post as deceptive. But Netanyahu Jr. might argue that the photo was an illustration photo, which did not purport to show actual Israeli-Arab voters. While in established printed and online media, there is a clear norm that illustration photos should be labeled as such, there is no such norm in social media. Additionally, it was unclear who stood behind the account of the original post that Netanyahu Jr. retweeted; thus, he could claim he was not personally responsible for the truth of the post.^13^

Unstable norms not only allow posters to evade responsibility, but also may negatively affect readers’ belief formation. Regina Rini (2017) argues that the cumulative weight of many likes and users’ repeated exposure to comforting views lower their skeptical defenses and make them believe fake news. She characterizes online posting as *bent testimony.* Rini writes:

The epistemic relationship between testifier and testimony [online] is ambiguous, as we haven’t yet settled on a norm whereby sharing entails assertion. Nevertheless, many of us treat social media sharing as if it were ordinary testimony, at least until something goes wrong (2017, E48).

Rini’s analysis is on the right track, but Rini wrongly treats all online posts and shares as testimony.^14^ Posting is *not* a distinct communicative act and cannot be analyzed according to a single standard. Online posts consist of a variety of communicative acts: assertive acts, directive acts; commissive acts, such as offering, promising, refusing, vowing and volunteering; expressive acts, such as congratulations; and declarative acts (Banikalef & Bataineh 2017). Only some of these types of posts admit of the analysis Rini provides.^15^

By contrast, as Emanuele Arielli (2018) argues, sharing, i.e., reposting or retweeting someone else’s post, where the original post or tweet is embedded within a new post, is a distinct communicative act:

while publicly posting a link to a content simply means to show that content to other viewers, explicitly sharing that link […] is a directive calling for other people’s attention and, at the same time, an assertion about the relevance or “shareworthiness” of what is pointed to […] *An act of sharing is therefore a speech act whose aim is to direct the attention of other people to a content, stating (or expressing) its shareworthiness* (Arielli, 2018, 253, emphasis is the origin).

We may thus formulate the norm of sharing as something like this:

*Norm-of-sharing*: share post x only if x is worthy of attention.^16^

This formulation is almost trivial, but nevertheless allows us to see why the norm is unstable. What is “worthy of attention” will depend on the particularities of the context, which, as noted, is rarely clear online. Indeed, it is often unclear why people want to draw others’ attention to a post. They may mean to communicate “I believe this,” “I don’t believe this!,” or “What do you think of this?.” We can add a meta-level: “I want you to believe this,” “I want you to believe I believe this,” “I want you to believe I am in the know,” and then the meta-meta: “I am trolling you” and “I want you to believe this, but not blame me for it.” The latter is arguably the case when former President Trump says or tweets “people are saying X” for some outrageous X.

Social identity maintenance is a prominent motivation for sharing. Sometimes a user shares a post because she has an overriding desire to be credited as the first who shared a piece of information in a group, in case it proves true (Gelfert, 2013). Another purpose of sharing, which explains why some people share posts without even reading beyond their title, is to maintain their reputation: to signal to seemingly like-minded people that they hold the same views, and reinforce their membership in the same group (Origgi, 2018, Ch. 3). People also share to appear helpful or gain social currency, namely, appear trendy, cool, and in the loop (Berger, 2013; Chakrabarti et al., 2018).

As in testimony, context collapse leads to an unstable norm of sharing, because different audiences have different standards for shareworthiness, and this in turn may lead to the spread of epistemically toxic content due to confusion about what makes the post worthy of attention. The norm of sharing, however, unlike the norm of testimony, is *not epistemic,* as it lacks checks on the epistemic standing of the content being shared. *This lack may further facilitate the spread of epistemically toxic content.*

While the norm of sharing is not epistemic, applying it to a specific context may have epistemic dimensions, around which there may be confusion and ambiguity, which may foster the spread of epistemically toxic content. If the original post (the one shared) consists of an assertion, the epistemic norm of assertion and users’ normative epistemic expectations that follow from it may transfer to the act of sharing. Namely, posters’ and readers’ judgment of its shareworthiness may depend on its perceived truth or validity.

Empirical research suggests that there are two competing norms of sharing (fake) news stories: a non-epistemic norm and an epistemic norm. The non-epistemic norm is something like “regardless whether post x is actually true or false, share x only if x would be interesting if x were in fact true.” The prevalence of this norm is backed by experiments that show that while people identify fake news as fake, they still share it if they judge it to be interesting if it is nevertheless true (Altay et al., 2021; cf. Pennycook et al., 2020). The second, competing epistemic norm is something like “share post x only if two conditions obtain: (1) x is true (or known or justified)^17^; (2) x is interesting.” In the second formulation, but not in the first, the post’s having a positive epistemic standing is a necessary condition for its being considered shareworthy. The prevalence of the second norm is backed by empirical research that shows that users are generally reluctant to share fake news because they—correctly—fear harming their online reputation (Altay et al., 2020).

Another epistemic dimension of the application of the norm of sharing to factive posts is ambiguity about who bears epistemic responsibility for it (recall *Election Photo* and see *Twitter Favoritism* below). As Wright (2021, 250) writes:

Re-posting without comment does not disambiguate the purpose of sharing. If the content of the re-posted article turns out to be false, the re-poster can deny responsibility [...] Regardless of the poster’s intention, re-posting puts audience members in touch with a source, and it provides the audience with at least some evidence that the re-poster trusts the site.

Empirical research suggests that here also two competing norms exist. One is “sharing is an endorsement”, while the other is “sharing is not an endorsement.” Which of these norms is correct and when is hotly debated in the courtroom in online defamation cases and outside it (Allen, 2014; Arielli, 2018; Marsili, 2021; Perry, 2021). Many people include the line “RT ≠ endorsement” (“retweets do not equal endorsement”) in their Twitter profile as a sort of blanket self-exculpation.

So far, we discussed sharing seemingly informative posts, and argued that people simultaneously apply two competing norms to them, a non-epistemic norm and an epistemic one. When people share, however, they “are not necessarily looking to inform others: they share stories (and pictures, and videos) to express themselves and broadcast their identity, affiliations, values, and norms” (Marwick, 2018, 505). Unfortunately, truth and validation may be a resource deployed in pursuit of these goals, but it is not the whole ballgame.

Much online behavior is best understood as seeking to produce a coherent story—whether true or not. Introne et al. (2018) recount an online community dedicated to the reality of Stargates, alien artifacts that create wormholes to other worlds. People in this community employ “unconventional epistemic strategies” to weave various pieces of evidence together into a coherent narrative. These strategies include “extrapolating from unconventional sources of evidence” including fictional accounts of Stargates; “rejection of rigorous research” that is not compatible with the group’s central dogma; and “unwarranted use of conventional knowledge,” such as deploying popular accounts of quantum mechanical effects as evidence of the plausibility of Stargates (2018, 2). Such epistemic strategies exist not just in fringe Internet communities. “Close reading,” a strategy familiar in religious studies, is sometimes applied to news, quotes or tweets from political figures, or legislation, to find evidence of deep truths or of partisan “fake news” (Marwick, 2018).

Introne et al. and Marwick argue that these strategies do not aim to assess the veracity of claims, but to produce verisimilitude with an existing worldview. People engage with social media to construct a predetermined narrative. Readers engage in motivated reasoning, taking advantage of the polysemy of posts, and employing strategies like the above to integrate the post into their understanding of the world, as in the following example.

**Fig. 1:**
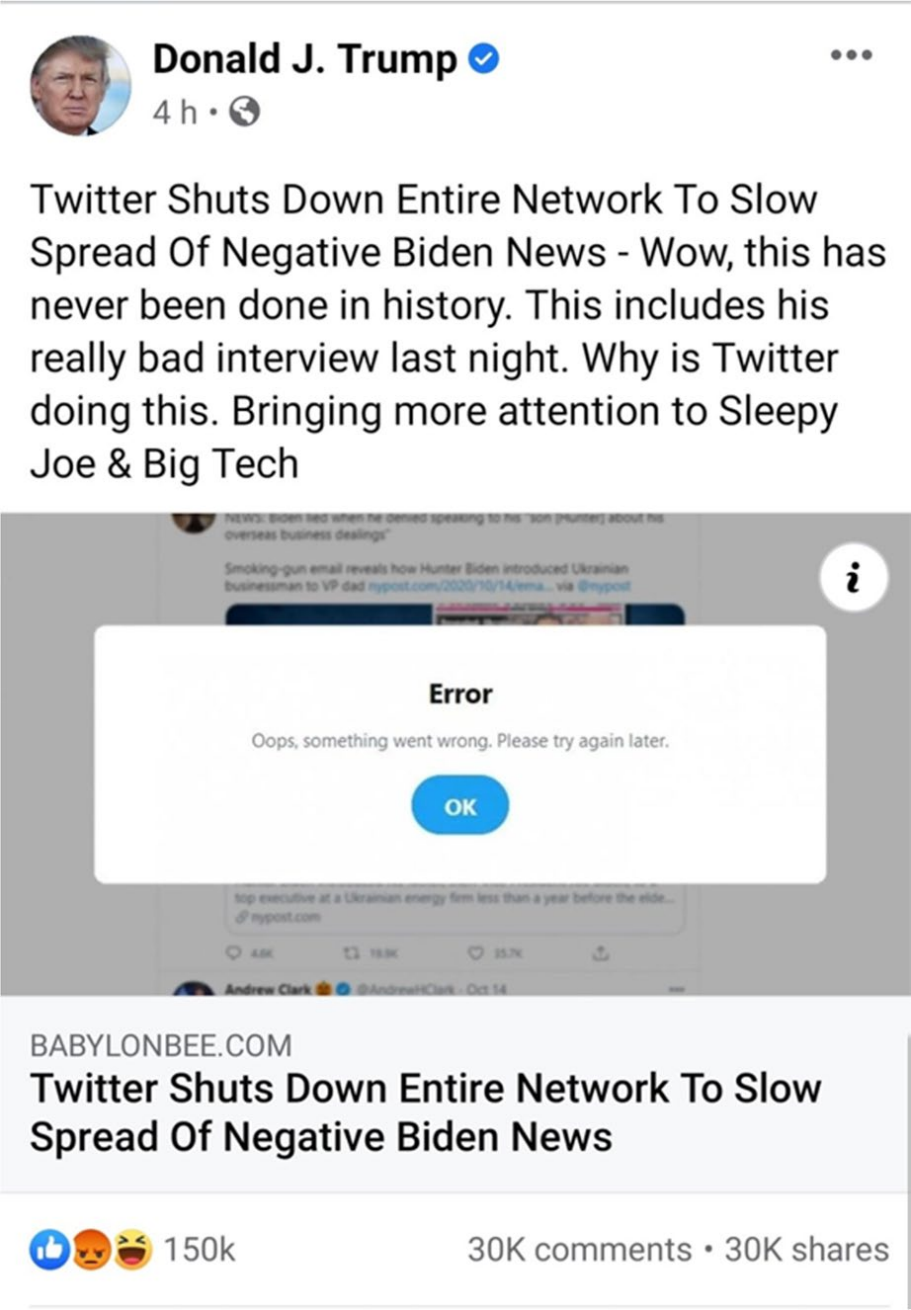
A Twitter post from US President Donald Trump resharing satirical content (October 16, 2020)

*Twitter favoritism*: A Twitter post from President Donald Trump reshares and extends content originating a post that describes favoritism toward one candidate for President by Twitter (Fig. 1). The link leads to the website *The Babylon Bee*, which looks like a legitimate news site. This post, however, is a satirical joke. To get the joke, the reader must correctly identify the context, namely, know or infer that *The Babylon Bee* is a satirical website. Many of the reactions and replies suggest that many in the audience, perhaps including the President, are unaware of the satire.

*Twitter favoritism* is illustrative. Lynch (2019, Ch. 2) singles out “outrage” as a primary driver of sharing. Indeed, some empirical evidence supports that high-arousal emotions, especially negative ones, are among six causes of virality (Berger 2013, Ch. 3).^18^ However, *Twitter favoritism* shows that outrage itself is not always the driver of virality; rather, outrage is sometimes a symptom of norm instability. That is, a reader’s outrage in this case is a result of her misunderstanding the claim as true. Moreover, people commented on Trump’s tweet noting that the story was a satirical fake, but their comments did not prevent others from expressing anger or sharing it as if it were true. This fact is consistent with the finding that many people share posts without reading beyond their title (Gabielkov et al., 2016), which Lynch explains as an expression of outrage, while Origgi (2018, Ch. 3) and Ganapini (2021) explain as a signal to like-minded people intended to reinforce their membership in the same social group. In any case, visible contextual information which would make clear that the post is satire, or enforceable norms which would require someone who shares a post in error to retract it could mitigate the spread of such a post (we expand about these suggestions in Sect. 5).

To conclude, the confusion around and instability of the norms of posting and sharing, which contribute to the spread of epistemically toxic content, are multilayered. Context collapse and diverse media ideologies among users are two contributing factors to this instability and confusion. Additionally, unlike the norm of assertion, the norm of sharing is non-epistemic. The lack of inherent epistemic checks in the norm contributes to the ease with which falsehoods and unsubstantiated claims spread online. Even when epistemic considerations play a role in users’ judgments of whether a post is shareworthy, they do so inconsistently.

This layered confusion and instability also result in users’ miscalibrated expectations about the truth and validity of the shared information, which in turn causes users to misevaluate posts’ and posters’ credibility, which in turn affects their belief formation and decision whether to reshare the post. It also creates uncertainty as to who is responsible for the shared post, which may allow posters to evade responsibility for information they share.

This is all a result of the interplay between users, platforms, and posts through technological and conceptual possibilities. These possibilities can be changed for the better through technology design and construction. A workable response to the challenges outlined above would need to address the warring incentives of sharing identity (for people), platform fixation on engagement crowding out truth, context collapse that derives from unstable media ideologies, the modular structure of the feed, and the ambiguous influence of the norm of sharing.

## Proposed solutions

A solution to the spread of epistemically toxic content online should address the arms race between sharing identity and platform engagement, context collapse, unstable media ideology, and the ambiguous influence of the norm of sharing. To be successful, a solution should acknowledge the autonomy of platform users to reinterpret platform-provisioned operations, and at the same time should resist silencing marginal voices. In this section, we hypothesize about possible interventions that should prove effective if our theoretical argument in this paper is right.^19^ Our proposed interventions involve both people and the platform.

An advantage of our proposed solution is that they do not involve content monitoring and censorship by platform providers, for which some scholars call (Gillespie, 2018; O’Conor & Weatherall 2019, 184). While we think that providers bear epistemic responsibility for the content that appears on their platforms, and we have elsewhere even argued that in some cases they should block certain kinds of content (Miller and Record, 2017); we also think that giving monopolistic mega-corporations the license to filter content for truth, hence the de facto power to determine what is true and false, should not be the first resort in addressing the problem. Another consideration against content monitoring is that Internet platforms have an inauspicious track record in transparently monitoring posts for offensive content or in giving users proper channels to appeal their decisions (Schwarz, 2019; Vaccaro et al., 2020). States similarly do not have a good track record regulating free flow of politically inconvenient information, and they may be even less trustworthy than private corporations (Coady, 2019; Origgi, 2013; Tufekci, 2017). Our approach nudges users towards epistemically responsible online sharing, and we think it should be generally preferred over top-down filtering-based solutions.^20^

While our approach is based on content monitoring by Internet platforms, it does require them to make changes in their interface that may result in decreased traffic, hence be against their commercial interests. We are not naive to think that all platforms will happily and voluntarily do so, but we think that they may cave in to public or regulatory pressure to do so, as they have done in the past.

### Set explicit norms

People can set explicit norms for spaces they have influence over, such as Facebook Groups, communities, or their own “walls.” Collectively, people can extend this idea to the single-context spaces of Twitter and Facebook, by modeling preferred behavior and coaching others.

*WhatsApp campaign:* This idea is captured in an advertisement campaign that Meta-owned WhatsApp launched on radio, television, and YouTube in India in 2018. WhatsApp is the most commonly used instant-messaging platform in India. The campaign included three short video advertisements, in which an ordinary user sees a post shared by another user, which contains a rumor. For example, a young woman is active in her family’s WhatsApp group. Her uncle shares an unsubstantiated rumor to the group, and she intervenes by calling him and asking him whether he had proof or just forwarded it. He says he does not, and that he is only sharing a post shared with him. She explains the potential violent consequences of sharing epistemically toxic posts and convinces him to leave the group that spread it to him. Other ads also mention the group admin’s possibility to block a user who shares epistemically toxic content, and individual users’ possibility to individually block such a user. According to WhatsApp, the scenarios in the videos were based on real use cases (Upadhyay, 2018).

WhatsApp released this campaign following several events in India in which people were lynched following widely shared WhatsApp posts accused them sex crimes, and after the Indian government demanded that WhatsApp take action. WhatsApp also changed its interface in India such that forwarded posts would be clearly marked as such, and a user could share a post to no more than five groups. While these measures can be criticized as being too little too late, setting explicit norms of use, specifically norms that debunk users’ common justifications or excuses for sharing harmful posts, encouraging users to follow and enforce them, and affording them technological possibilities to do so, are still right measures to take to mitigate the spread of epistemically toxic content.^21^ Groups and online communities can similarly be encouraged to set appropriate local norms against sharing harmful posts.

Gershon notes that people evaluate media based on similarity to other media or face-to-face conversation. People transfer expectations from one medium to another, so conditioning good behavior in well-defined contexts is likely to influence peoples’ behavior outside of those contexts as well. Another example is:

*Hearts and thumbs*: in the pivot to online work during the coronavirus outbreak in 2020, a workplace adopts Microsoft Teams, which includes a chat function with two reactions, heart and thumbs-up. The meanings are ambiguous: does heart mean love or support? Does it refer to the content of the post or the poster? A team member sends a message to the entire team: “Let’s agree that thumbs up means you have read the message. Heart means you endorse the contents.”^22^

Creating stable norms around responsibility for the content of posts (*WhatsApp campaign*) and the meaning of ambiguous interface elements (*Hearts and thumbs*) can help fight epistemically toxic content. There is a lot platforms can do to help. They can add new reactions and provision managers of groups with tools to moderate activities within the community. As Lynch (2019, 48) suggests, they can add reactions that consist of normative epistemic evaluation of the post, such as “justified by the evidence,” “not justified by the evidence,” and “need more information.” (We are not naive to think, however, that they will do so enthusiastically.) On Reddit, each channel has its own norms, allowing different communities to establish different media ideologies and norms.

### Solution 2: context, context, and more context

Platforms can change posts and operations in ways that will nudge people toward preferred behaviors. They can make it easier to report bad behavior, change default behaviors, or they can add friction to operations associated with problematic behavior online. Platforms could modify the algorithm to place related posts adjacent to one another in the feed, thus adding some context back into the modular feed.

Platforms can build easier ways for people to add context to their posts, especially reposts. For example, the sharing interface could make it as easy to signal agreement or support as to simply reshare. In its early days, Facebook had one reaction button: “like.” People have long asked for additional reactions, such as “dislike” and “sarcasm.” In February 2016, it added five new reactions: love, haha, wow, sad, and angry. These represent some of the most popular reactions people use, but it is only a fraction of the many reactions that would be needed to capture a fuller range of human emotion. In April 2020, with the first wave of COVID-19, Facebook added a seventh reaction: “care” (Stinson, 2016; Lyles, 2020).

The default behavior of the share interface on Facebook is to share the original post with no place for the sharer to add her thoughts about it or her reasons to share. To add such context, the user must reedit the post on her profile. When a post includes an external link, sometimes (depending on its visibility settings) only the link is shared, without the original poster’s comments, which may be the very content the sharer wants to share. Facebook should take an opposite approach. It should nudge, even require, users to write some words contextualizing the share. Even just embedding a date in a post can prevent obsolete news stories from resurfacing as relevant to current events.^23^

A more ambitious approach is bringing community governance to social media. Platforms provide means for marginal voices of finding an audience, but they are centrally administered. With an algorithmic feed designed for “engagement,” the result is a media environment that favors extremes, not diversity. The apparent choice is whether we want to hear all the voices but have no common ground, or return to a common ground selected by elites (or worse, algorithms). But this is a false dilemma. The problem with social media is not that algorithms are worse curators than elites, but that we are delegating curation in the first place. Platforms have a significant role to play in helping people curate their own information environments. Platforms can slow the feedback loop enough to let people react with more consideration, and platforms can provide tools for users to govern their own communities, their own feed, and how their own posts appear to others.

## Conclusion

The circulation of epistemically toxic content online has been widely misunderstood. Close attention to the media environment as a whole, including people’s behaviors, posts’ content, and the operations platforms afford, has allowed us to diagnose the problem and construct workable responses. Context collapse and unstable media ideologies are significant obstacles to the successful fulfillment of an epistemic norm, and to recipients’ making correct inferences from other people’s posts. Sharing is a highly under-regulated speech act, which leads to faulty belief formation and credibility assignment, and allows posters to evade responsibility for harmful posts. These are all catalysts for the spread of epistemically toxic context online. This spread can be effectively fought by making norms of sharing more explicit, and by encouraging or requiring users to add context to their posts.

It might be thought that attending too closely to the details of a specific technology or behavior is a waste, since they change so quickly. We disagree. Changes in technology and behavior have always changed our epistemic landscape. A central lesson of our analysis is that there is no escaping the need to attend closely to what actually happens.

## Data Availability

Data sharing not applicable to this article as no datasets were generated or analyzed during the current study.

## Footnotes

1 Anderau (2021) provides a thorough review of definitions of “fake news”; Coady (2019; 2021) criticizes the term fake news as serving the function of silencing views that are inconvenient to the hegemony.

2 Phillips and Milner (2021) and Frost-Arnold (2021a) fruitfully explore the metaphor of a polluted media landscape.

3 Marwick (2018), Phillips and Milner (2018), and Ben Thompson of stratechery.com were important influences for this list.

4 There is not universal agreement on this point. But see Alfano et al. (2021) for a recent discussion of the mechanisms involved.

5 Thanks to a reviewer for helping us moderate this claim. As Coady (2019) discusses, the distinction between “traditional” and “online” or “social” media are often overblown. Nevertheless, we think the short feedback loop of social media algorithms does make an important difference.

6 See Marwick (2018) for further discussion of participatory culture.

7 This pattern is reminiscent of one described by social constructivists of technology in terms of “interpretive flexibility” and “closure” (Bijker et al., 1987).

8 Gershon conducted her original research in 2008, when she studied undergraduate students’ romantic breakups on social media. In 2018, she followed up on her research, and found out that media ideologies among undergraduate students have largely stabilized. One factor responsible for this stabilization is a coming of age of the media. Specifically, different platforms (Snapchat, Instagram, etc.) provide similar affordances, which allow for a shared interpretation of the meaning of the messages sent through them. On the other hand, when different media provide different affordances, a sender’s deliberate choice of one medium over another provides the receiver further contextual information that helps her better interpret the message (Gershon 2020). It seems that in 2018, Gershon is less committed than in 2008 to the view that media ideologies are largely detached from the material capacities of a medium, which are exemplified in the action possibilities it affords. We see her revised stance as supporting our general approach in this paper.

9 Frost-Arnold (2021b) has independently developed a similar analysis of the epistemic pitfalls of context collapse. For more cases, see Ronson (2015), Detel (2013), and Poerksen & Detel (2014).

10 Screenshots of the correspondence are available at twitter.com/aradboaz/‌status/‌129039‌1598‌7910‌00071.

11 There is a debate whether telling covers all cases of testimony, and whether technologies can give testimony or only humans. See Freiman and Miller (2020).

12 See Kneer (2018) versus Turri (2020).

13 Screenshots of the post and the revelation that the photo is from Turkey are available at twitter.com/‌SrugimNews/‌status/‌1173924299704590336.

14 In a footnote, Rini (2017, E60, fn. 10) acknowledges this difficulty.

15 Cf. Marin: “For users, the norms for sharing are clearly different than those of posting, yet what these norms are is hard to tell” (2021, 365).

16 We thank Sandy Goldberg for helping us reach this formulation.

17 We hypothesize that the exact condition is derivable from the norm of assertion that operates in that context.

18 Berger’s theory, however, cannot explain all cases of viral content. For example, in 2019, a photo of an ordinary egg with the caption “Let's set a world record together and get the most liked post on Instagram” received 18.4 million likes within 10 days, and became most-liked Instagram post of all time. An alternative theory that can potentially explain this case is Nahon and Hemsley (2013) gatekeeping theory of virality. A gatekeeper is an agent who can transfer information to others or block it. According to this theory, content becomes viral when it triggers a chain reaction among gatekeepers of two kinds: (1) a gatekeeper with many followers and (2) a gatekeeper with diverse followers in separate social clusters (2013, Ch. 3). While certain attributes of the content, such as its being remarkable or outrageous, are a catalyst of virality, they are neither necessary nor sufficient for its becoming viral (2013, 61–67). The primary factor that makes content viral is who spreads it. Moreover, a gatekeeper’s perceived trustworthiness on a given message is relevant to users’ decision to pass it on (2013, 49).

19 Empirical tests of our hypotheses are in progress. Several experiments that test the efficacy of concrete interventions derived from the theoretical framework developed in this paper have been conducted. Their results seem promising. They are being prepared for publication.

20 Another approach is to outsource content monitoring to independent fact-checking organizations. We think that this approach may be effective against certain types of posts, such as widely-shared scams or blunt falsehoods, but it has its limitations. First, it has been argued that fact-checking organizations unjustifiably claim to conclusively verify statements that are inherently uncertain and to make decisive judgments regarding claims that involve legitimate semantic ambiguity (Uscinski & Butler 2013; for rejoinder, see Amazeen 2015). Second, heavy reliance on fact-checking reverts new media to epistemic gatekeeping practices of old media, which may silence legitimate voices of members of underrepresented and marginalized groups. Third, and most important for this paper, fact checking is mostly effective against widely shared posts or posts in widely followed pages, but fact-checking organizations do not have the resources to monitor the vast amount of toxic posts made and shared by regular users, which our approach specifically targets. Last, some think that fact-checking and content-filtering can be automated. We remain skeptic, but addressing this issue exceeds the scope of this paper. For some critical discussion, see Gelfert (2021).

21 Unpublished experimental results suggest that such measures are indeed effective.

22 As a reviewer has pointed out, formal norms like this are susceptible to dissent or trolling. Nevertheless, we think a suitably empowered community can enforce norms even in the face of opposition. Our intention is to suggest changes to both norms and the platform-provisioned operations that can work in concert to address this concern.

23 In the lead up to the 2020 US election, Twitter changed from a choice of retweet and quote-tweet to a default of quote-tweet, making it one click easier to add context to a share. At the time of this writing, Twitter offers retweet and quote-tweet as options. We are cautiously optimistic that interventions such as this will continue to nudge users toward more thoughtful interactions.
